# Pembrolizumab plus azacitidine in patients with chemotherapy refractory metastatic colorectal cancer: a single-arm phase 2 trial and correlative biomarker analysis

**DOI:** 10.1186/s13148-021-01226-y

**Published:** 2022-01-06

**Authors:** Chaoyuan Kuang, Yongseok Park, Ryan C. Augustin, Yan Lin, Douglas J. Hartman, Lindsey Seigh, Reetesh K. Pai, Weijing Sun, Nathan Bahary, James Ohr, John C. Rhee, Stanley M. Marks, H. Scott Beasley, Yongli Shuai, James G. Herman, Hassane M. Zarour, Edward Chu, James J. Lee, Anuradha Krishnamurthy

**Affiliations:** 1 UPMC Hillman Cancer Center, Pittsburgh, USA; 2 Division of Hematology-Oncology, Department of Medicine, School of Medicine, University of Pittsburgh, UPMC Cancer Pavilion, 5150 Centre Avenue, Room 463, Pittsburgh, PA 15232 USA; 3 Hillman Cancer Center Cancer Therapeutics Program, Pittsburgh, USA; 4 Present Address: Albert Einstein Cancer Center, Montefiore Einstein Cancer Center, Albert Einstein College of Medicine, 1300 Morris Park Avenue, Chanin 628, Bronx, NY 10461 USA; 5 Graduate School of Public Health, University of Pittsburgh, Pittsburgh, USA; 6 Division of General Internal Medicine, Department of Medicine, School of Medicine, University of Pittsburgh, Pittsburgh, USA; 7 Department of Pathology, School of Medicine, University of Pittsburgh, Pittsburgh, USA; 8 University of Kansas Cancer Center, Westwood, USA; 9 AHN Cancer Institute, Pittsburgh, USA; 10 Hillman Cancer Center Cancer Epidemiology and Prevention Program, Pittsburgh, USA; 11 Hillman Cancer Center Cancer Immunology and Immunotherapy Program, Pittsburgh, USA

**Keywords:** Colorectal cancer, Mismatch repair proficient, Immunotherapy, DNA methyltransferase inhibitor, Epigenetic therapy, Azacitidine, Pembrolizumab, PD-1, PD-L1

## Abstract

**Background:**

DNA mismatch repair proficient (pMMR) metastatic colorectal cancer (mCRC) is not responsive to pembrolizumab monotherapy. DNA methyltransferase inhibitors can promote antitumor immune responses. This clinical trial investigated whether concurrent treatment with azacitidine enhances the antitumor activity of pembrolizumab in mCRC.

**Methods:**

We conducted a phase 2 single-arm trial evaluating activity and tolerability of pembrolizumab plus azacitidine in patients with chemotherapy-refractory mCRC (NCT02260440). Patients received pembrolizumab 200 mg IV on day 1 and azacitidine 100 mg SQ on days 1–5, every 3 weeks. A low fixed dose of azacitidine was chosen in order to reduce the possibility of a direct cytotoxic effect of the drug, since the main focus of this study was to investigate its potential immunomodulatory effect. The primary endpoint of this study was overall response rate (ORR) using RECIST v1.1., and secondary endpoints were progression-free survival (PFS) and overall survival (OS). Tumor tissue was collected pre- and on-treatment for correlative studies.

**Results:**

Thirty chemotherapy-refractory patients received a median of three cycles of therapy. One patient achieved partial response (PR), and one patient had stable disease (SD) as best confirmed response. The ORR was 3%, median PFS was 1.9 months, and median OS was 6.3 months. The combination regimen was well-tolerated, and 96% of treatment-related adverse events (TRAEs) were grade 1/2. This trial was terminated prior to the accrual target of 40 patients due to lack of clinical efficacy. DNA methylation on-treatment as compared to pre-treatment decreased genome wide in 10 of 15 patients with paired biopsies and was significantly lower in gene promoter regions after treatment. These promoter demethylated genes represented a higher proportion of upregulated genes, including several immune gene sets, endogenous retroviral elements, and cancer-testis antigens. CD8^+^ TIL density trended higher on-treatment compared to pre-treatment. Higher CD8^+^ TIL density at baseline was associated with greater likelihood of benefit from treatment. On-treatment tumor demethylation correlated with the increases in tumor CD8^+^ TIL density.

**Conclusions:**

The combination of pembrolizumab and azacitidine is safe and tolerable with modest clinical activity in the treatment for chemotherapy-refractory mCRC. Correlative studies suggest that tumor DNA demethylation and immunomodulation occurs. An association between tumor DNA demethylation and tumor-immune modulation suggests immune modulation and may result from treatment with azacitidine.

*Trial registration* ClinicalTrials.gov, NCT02260440. Registered 9 October 2014, https://clinicaltrials.gov/ct2/show/NCT02260440.

**Supplementary Information:**

The online version contains supplementary material available at 10.1186/s13148-021-01226-y.

## Background

Colorectal cancer (CRC) remains a major public health problem in the US and globally [1]. Metastatic CRC (mCRC) is usually associated with poor prognosis, with 5-year survival rates in the 5–8% range. Chemotherapy remains the backbone of treatment for patients with mCRC. The median OS with best supportive care alone in patients with mCRC after progression with standard treatment options is historically only about 5 months, and survival is improved by only about 2 months with the addition of either regorafenib or TAS-102 [2, 3]. There is clearly a significant unmet need for new treatment regimens that can provide durable disease control in mCRC.

Programmed cell death 1 (PD-1) pathway blockade enhances tumor antigen-specific CD8^+^ T cell responses [4]. The targeting of this immune checkpoint pathway with monoclonal antibodies to either the PD-1 receptor or to PD-L1 ligand has led to highly durable tumor response and minimal toxicity in the treatment of several tumor types, including DNA mismatch repair deficient (dMMR) mCRC [5–7]. However, to date, PD-1 blockade monotherapy has not demonstrated meaningful antitumor activity in mismatch repair proficient (pMMR) mCRC [8, 9]. Possible reasons for the lack of a robust antitumor response to PD-1 blockade in pMMR mCRC include the relative absence of infiltrating CD8^+^ T cells in tumors [10, 11] and the lower tumor mutation burden [12, 13]. These findings suggest it may be necessary to combine PD-1 blockade with other therapeutic approaches aimed at increasing the immunogenicity of CRC tumors.

Azacitidine is a DNA methyltransferase inhibitor (DNMTi) that decreases DNA methylation, allowing re-expression of genes previously silenced by DNA hypermethylation, including tumor-associated antigens and endogenous retroviruses (ERVs) [14, 15]. This agent was initially approved by the US FDA for the treatment of patients with myelodysplastic syndrome [16–18]. Previous studies have demonstrated that epigenetic modulation by DNMTi modifies the expression of genes related to innate immunity, adaptive immunity, and immune evasion in tumor tissues [19–21]. Chou et al. reported that decitabine, a DNMTi, induced expression of NY-ESO-1 and other cancer-testis antigens (CTAs) in CRC cells both in vitro and in vivo [22]. Kim et al. reported that epigenetic modulation with azacitidine and entinostat, a histone deacetylase inhibitor (HDACi), markedly improved the antitumor activity of checkpoint inhibitors in a murine CT26 pMMR CRC model. Of note, the antitumor activity was mainly due to the inhibition of myeloid-derived suppressor cells (MDSCs) by the combined epigenetic modulation [23]. Yu et al. reported that treatment of murine CT26 CRC tumors with azacitidine induced an immunomodulatory response marked by increased intratumoral CD3^+^, CD4^+^, and CD8^+^ T cells [24]. Taken together, these studies suggest that treatment with a DNMTi may enhance the antitumor immune response by promoting increased tumor-infiltrating lymphocytes (TILs), although specific mechanisms by which this occurs have not been established. This clinical study was undertaken to evaluate the antitumor activity resulting from combining the PD-1 checkpoint inhibitor pembrolizumab with epigenetic modulation by azacitidine in patients with progressive, refractory mCRC with no further standard treatment options.

## Methods

### Patients

Eligible patients were ≥ 18 years of age with histologically confirmed mCRC that had been treated with currently approved standard therapies, including fluoropyrimidine-, oxaliplatin- and irinotecan-based chemotherapy, anti-VEGF therapy, and, if RAS wild-type, anti-EGFR therapy. Other major eligibility criteria included Eastern Cooperative Oncology Group performance status of 0 or 1 and adequate laboratory values (Additional file 1: protocol). Disease was measured based on Response Evaluation Criteria in Solid Tumors (RECIST) 1.1 criteria. Exclusion criteria included diagnosis of immunodeficiency; receiving systemic steroid therapy or any other form of immunosuppressive therapy within 7 days prior to cycle 1 day 1; chemotherapy, targeted agent, or radiation therapy within 4 weeks prior to cycle 1 day 1; known active CNS metastases and/or carcinomatous meningitis; active interstitial lung disease or pneumonitis; prior therapy with immune checkpoint inhibitors; and known history of HIV or active HBV or HCV infection.

### Study design

This was an investigator-initiated, phase 2, open-label, single-center trial of pembrolizumab in combination with azacitidine in patients with chemotherapy-refractory mCRC (*Trial registration: ClinicalTrials.gov, NCT02260440. Registered 9 October 2014, *https://clinicaltrials.gov/ct2/show/NCT02260440). The primary objective of this study was to evaluate the antitumor activity of pembrolizumab in combination with azacitidine in patients with previously treated mCRC without further standard treatment options, and the primary endpoint was overall response rate (ORR) using RECIST version 1.1. The secondary objectives included assessment of safety and tolerability, progression-free survival (PFS), and overall survival (OS). Exploratory objectives were to evaluate whether treatment with the combination of azacitidine and pembrolizumab was able to induce expression of CTAs and immune-checkpoint proteins in tumor tissue, global and targeted methylation of relevant genes, and immune profiling for specificity against relevant antigen targets.

Pembrolizumab was administered at the fixed dose of 200 mg by intravenous (IV) infusion on day 1 of each cycle on 21-day cycles (Additional file 2: Fig. S1). Azacitidine 100 mg was administered daily by subcutaneous (SC) injection on days 1–5 of each cycle on 21-day cycles. The study treatment was continued until progression, withdrawal of consent, or intolerance. Safety evaluations occurred weekly, with treatment-related adverse events (TRAEs) graded in accordance with the National Cancer Institute’s Common Terminology Criteria for Adverse Events, version 4.0. Treatment continuation beyond first radiographic progression was permitted at the discretion of the investigator if it was determined that patient safety was not at risk.

Computed tomography (CT) scans (chest, abdomen, pelvis, and other involved areas) were assessed at baseline and every 8 weeks until progression. RECIST version 1.1 was used to categorize overall best response as a confirmed complete response or partial response (PR), stable disease (SD), or disease progression (PD), with progression-free survival (PFS) measured from treatment initiation until PD or death from any cause, and overall survival (OS) from treatment initiation to death from any cause. PFS and OS were estimated by Kaplan–Meier methods and other results by descriptive statistics. All patients who received at least one dose of either pembrolizumab or azacitidine were included in the safety and efficacy analysis.

Patient characteristics, including *KRAS* mutation, *BRAF* mutation, and MMR status, were determined by chart review of clinical testing. At the time this study was designed, the sensitivity of dMMR CRC to immune checkpoint blockade was not well-described yet. Therefore, this study plan did not include MMR status as a stratification or inclusion criterium.

### Statistical analysis

This trial followed Thall, Simon, and Estey’s design to simultaneously and continuously monitor the efficacy and toxicity [25]. Patient enrollment was to be stopped if there was sufficient evidence of either futility or excess toxicity. The ORR of the currently available treatment for chemo-refractory mCRC is < 5% [2, 3]. Based on the existing data at the time, we estimated the severe toxicity rate for either single treatment would be less than 20% [26]. We considered the combination to be worthy of further investigation if the objective response rate was 20% or higher and the toxicity rate was 30% or lower. The target recruitment goal was 40 patients. Based on our study design, accrual must stop if zero patients responded among 10–19 accrued patients, if one or fewer patient responded among 20–30 accrued patients, if two or fewer patients responded among 31–39 accrued patients, and if three or fewer patients responded among 40 accrued patients. For survival analyses, pointwise 95% confidence intervals were determined for the Kaplan–Meier survival analyses using the Greenwood estimator. The 95% exact confidence interval of response rate was determined using the Clopper–Pearson method.

### Biomarker studies

#### Immunohistochemistry

Descriptive assessments by IHC were pre-planned as a part of this trial. Programmed-death ligand 1 (PD-L1) expression was determined using fresh formalin-fixed, paraffin-embedded tissue sectioned at 4 microns, with a proprietary assay developed at QualTek Molecular Laboratories (Newtown, PA). The assay used the 22C3 anti–PD-L1 murine monoclonal antibody (Merck Research Laboratories; Palo Alto, CA) as the primary reagent, and Envision FLEX + (Dako; Carpinteria, CA) ancillary reagents for antigen retrieval, as the secondary antibody, and for chromogenic development. Staining was automated on the Techmate 500, which is no longer commercially available. Scoring was performed by a board-certified pathologist. Modified H-score was utilized for analysis of PD-L1 expression. See Additional file 1: methods for details.

#### DNA-methylation profiling

Assessment of global DNA methylation was pre-planned as a part of this trial. Quantitative comparison of global gene promoter DNA methylation and transcription factor binding sites (TFBS) was performed post hoc. Methylation analysis required 500 ng of isolated DNA and was performed on the Illumina Infinium EPIC array to obtain genome-wide methylation data (Illumina; San Diego, CA), according to the manufacturer’s instructions. Raw data files were preprocessed using *minifi*. DNA-methylation data were normalized by performing background correction and dye bias correction. Methylation probes containing common SNPs or mapping to chromosomes X and Y were excluded. Pre- versus on-treatment comparison was performed using Wilcoxon signed-rank test.

#### RNA sequencing

RNA sequencing analysis was performed post hoc. RNA was extracted from FFPE blocks using the AllPrep kit (Qiagen #80284), indexed libraries were prepared using the TruSeq RNA Library Prep for Enrichment (Illumina #20020189), and TruSeq RNA UD Indices (Illumina #20022371) according to manufacturers’ instructions. Libraries were normalized and sequenced by the UPMC Genomics Core using an Illumina Novaseq sequencer at 22 million paired-end reads per library. RNA counts were normalized and filtered using the R/Bioconductor packages edgeR and limma as described by Law et al. [27] A differential expression analysis was then assessed between pre- and post-treatment samples. Both gene set enrichment analysis (GSEA) and gene set variation analysis (GSVA) were performed upon the gene sets of interest (e.g., ERV, CTA, and various immune signatures). Pre- versus post-expression analysis was performed using Wilcoxon signed-rank test. All genomic analyses were performed using R (v4.0.3).

#### CD8^+^ T cell density quantification

CD8^+^ T cell density was performed post-hoc. Slides that had been stained with a CD8 antibody were scanned at 20 × magnification using an Aperio CS2 (Leica BioSystems, Buffalo Grove, IL). A freehand annotation was placed around the tissue fragments (core biopsy) and automated image analysis using a modified Aperio nuclear v9 algorithm (Leica BioSystems, Buffalo Grove, IL) was performed as previously described [28]. Briefly, this image analysis algorithm identifies cells with brown chromogen within the cell’s cytoplasm. The intensity of the chromogen is measured on a 0–3+ scale; Cells with intensities of 1+, 2+ and 3+ are considered positively identified cells. The density of the CD8 cells within the tissue section is determined by dividing the number of cells by the area analyzed. The pre-treatment and on-treatment specimens were analyzed in a similar fashion. Pre- versus on-treatment samples were compared using Wilcoxon signed-rank test. Benefiter versus non-benefiter samples were compared using Wilcoxon Rank-Sum test. Correlation between changes in TIL density and changes in methylation was calculated using the Spearman non-parametric method, with one-tailed p value. All statistical calculations for correlative data were performed using GraphPad Prism version 9.0.0.

## Results

### Patients

Thirty-one patients were enrolled between January 2015 and January 2016, with 30 patients receiving active treatment. One patient was enrolled but experienced disease progression prior to starting treatment, and therefore was excluded from safety and clinical efficacy analysis. Patient demographics and baseline characteristics are listed in Table 1. Twenty-one patients were confirmed to be pMMR, the MMR status of eight patients were unknown. This trial was launched prior to the discovery showing that PD-1 blockade is highly effective for patients with dMMR CRC, thus this trial enrolled one dMMR patient. Of the eight patients with unknown MMR status, five harbored *KRAS*-mutant tumors. A total of 17 patients had *KRAS*-mutant tumors. One patient’s tumor carried the *BRAF* V600E mutation and was pMMR. All 30 patients had expired at the time of manuscript’s preparation. The median duration of treatment was 3 cycles (range, 1–8 cycles). Ten of 30 patients were unable to complete the first 3 cycles due to rapid symptomatic tumor progression and were never restaged nor had on-treatment biopsies obtained, but were included as part of the safety and efficacy analysis. Patient enrollment was stopped prematurely due to futility, in accordance with predetermined Bayesian stopping rules (see Additional file 1: protocol).

**Table 1:** Patient demographics and baseline characteristics

Patient characteristics	N (30)	%
Median age, years (range)	61	(30–79)
Sex		
Male	17	57
Female	13	43
ECOG Performance status		
0	18	60
1	12	40
MMR status		
pMMR	21	70
dMMR	1	3
Unknown	8	27
*KRAS* Mutation status		
Mutated	17	57
Wild type	11	37
Unknown	2	7
*BRAF* Mutation status		
V600E	1	3
Wild type	20	67
Unknown	9	30
Median lines of prior systemic therapy, lines (range)	3	(2–5)
2	14	47
3	10	33
4	3	10
5	3	10
Previous systemic therapy		
FOLFOX + Bevacizumab	21	70
FOLFIRI + Bevacizumab	18	60
Regorafenib	10	33
FOLFOX	6	20
FOLFIRI + Ziv-aflibercept	4	13
FOLFIRI + Cetuximab	3	30
Other	18	60

### Safety

TRAEs were reported in 63% of patients (19/30), with nearly all of the TRAEs being grade 1 or 2 (96%). Frequent TRAEs of any grade occurring in > 10% of patients (*N* = 30) included: nausea (27%), fever (23%), anemia (10%), leukopenia (10%), constipation (10%), and ALT increase (17%). All TRAEs could be attributed to one of the two agents based on the experience of monotherapy treatment. No grade 4 or higher adverse events were observed. Our safety data suggest that this combination treatment with subcutaneous azacitidine and pembrolizumab is well-tolerated and not associated with additive or unforeseen toxicities (Table 2).

**Table 2:** Treatment-related adverse events

Treatment-related adverse events (%, N = 30)	Grade 1	Grade 2	Grade 3
General			
Nausea	27	0	0
Fatigue	17	7	0
Fever	3	0	0
Emesis	3	0	0
Dehydration	3	0	0
Anorexia	7	0	0
Cough	3	0	0
Pain	3	0	0
Hypoalbuminemia	3	3	0
Hematologic			
Anemia	7	0	3
Leukopenia	7	3	0
Lymphopenia	3	7	0
Neutropenia	7	0	0
Thrombocytopenia	3	0	0
Gastrointestinal			
Constipation	0	10	0
Flatulence	3	0	0
Diarrhea	3	0	0
Hepatic			
ALT elevation	10	3	3
Alkaline phosphatase elevation	3	0	0
AST elevation	3	0	3
Dermatologic			
Injection site reaction	7	0	0
Ecchymosis	3	0	0
Rash	3	0	0
Others			
Renal dysfunction	0	3	0
Hypoxia	3	0	0
Bradycardia	3	0	0
Headache	3	0	0
Stroke	3	0	0

### Clinical efficacy

**Fig. 1:**
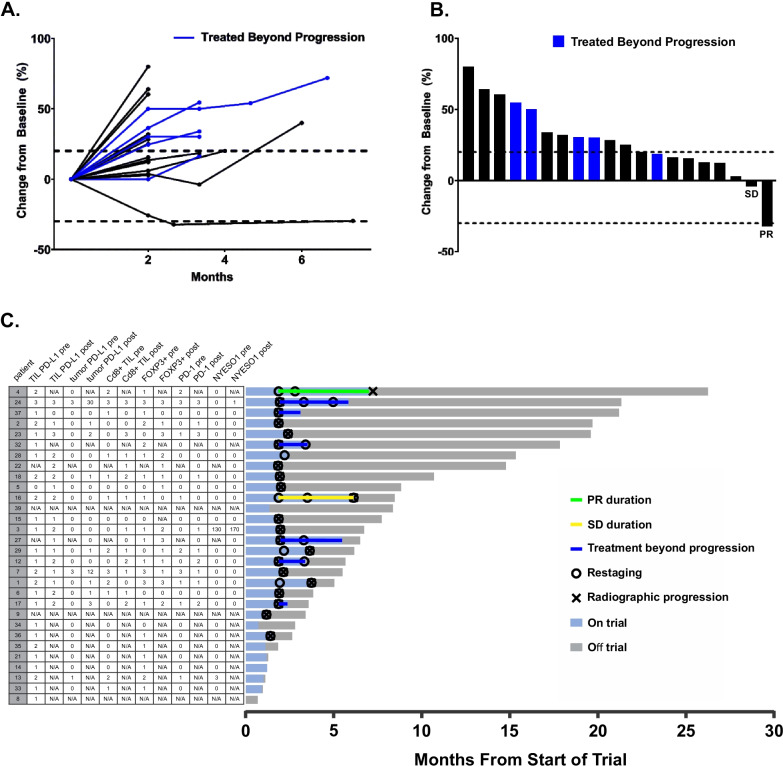
Activity and immune biomarkers of pembrolizumab in combination with azacitidine. **A** Percent change from baseline by RECIST 1.1 at each restaging time point by computed tomography. **B** Best response seen by RECIST 1.1. **C** Swimmers plot of treatment course of each patient in the intent to treat population. Immunohistochemistry of immune biomarkers in pre-treatment and on-treatment samples are indicated by numbers in adjacent table. The following were scored based on overall staining strength on scale of + 1 to + 3: TIL PD-L1 pre, TIL PD-L1 post, CD8^+^ TIL pre, CD8^+^ TIL post, FOXP3^+^ pre, FOXP3^+^ post, PD-1 pre, PD-1 post. The following were scored using a modified H-score (0 to 300): tumor PD-L1 pre, tumor PD-L1 post, NY-ESO-1. N/A is not assessed due to either no evaluable tumor or no biopsy obtained. CD8^+^ TIL and PD-1 indicate only intratumoral staining, does not include stromal or tumor/stroma interface staining. FOXP3^+^ are all intratumoral or stromal FOXP3^+^ Treg, although almost every sample had FOXP3 at the tumor/stroma interface. See Additional file 1: methods for additional details

Of the 30 treated patients, ten did not have post-baseline radiologic assessments for tumor response, as they withdrew from study due to either disease-related complications or disease progression. The one dMMR patient was among this group who was not restaged. One patient with pMMR mCRC achieved a confirmed partial response (PR) after 4 cycles, and 1 patient had stable disease (SD) as the best confirmed response (Fig. 1). Overall response (ORR) by RECIST1.1 was 3% (95% CI, 0–17%). Five patients with PD at the first restaging at the end of cycle 3 continued on study therapy, and 2 of these 5 patients had temporary stabilization of tumor progression on subsequent cycles (Fig. 1A). In total, 4 of 30 patients appeared to derive clinical benefit from therapy. Upon retrospective review, one additional patient was determined to have been treated beyond progression, as their first restaging scan had a new indeterminate hepatic lesion, which later progressed on imaging scans. Several patients were initially found to have SD as their best measured response on first restaging also had new lesions or symptomatic progression, and were thus designated as PD (Fig. 1B). Overall, this trial failed to meet its primary endpoint of ORR, which had been set at 20%.

The one patient with a partial response was a 52-year-old male with pMMR mCRC, and he was heavily pretreated with four prior lines of systemic chemotherapy. His tumor was determined to be pMMR by IHC with four markers of MLH1, PMS2, MSH2, and MSH6, and harbored a *KRAS* G12D mutation. When the patient was first enrolled in the study, the sites of disease included metastases in the mediastinal lymph nodes, bilateral lungs, right adrenal gland, retroperitoneal lymph nodes, and pelvic mesentery. All sites of disease demonstrated radiographic decreases in tumor size while on treatment (Additional file 2: Fig. S2). He received a total of 7 cycles of therapy on trial and was removed after confirmation of TRAE grade 2 acute tubular injury on renal biopsy. Following progression on study treatment, he received the oral fluoropyrimidine TAS-102, experienced slow progression of disease, and survived for nearly two more years. The one patient with stable disease was a 57-year-old male who had received 3 prior lines of chemotherapy and had metastatic disease involving the liver, abdominal wall, and pelvic omentum at the time of starting trial therapy. His tumor was pMMR by IHC and harbored a *KRAS* G12L mutation. He received eight cycles of therapy before experiencing progression of all measurable sites, as well as new disease in the sacrum. He received palliative radiation after stopping trial therapy, and succumbed approximately 50 days after coming off of study.

**Fig. 2:**
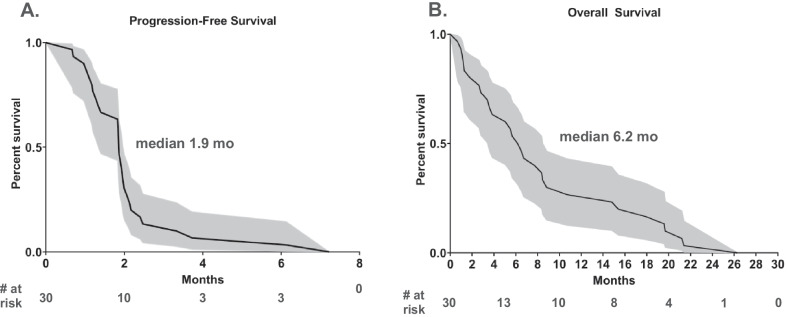
Patient survival data. **A** Progression-free survival (PFS) and **B** Overall survival (OS). Gray area demarcates 95% confidence intervals. All survival data are uncensored

The median PFS among all 30 patients was 1.9 months (95% CI, 1.3–2.0 months), and the median OS was 6.2 months (95% CI, 3.4–8.5 months) (Fig. 2). The median duration of treatment was short at 2.2 months (95% CI, 1.4–2.6 months). The longest surviving patient was the only responder on trial. The second and third longest surviving patients experienced PD on first restaging; however, both were continued on trial due to favorable tolerability. Both of these patients received additional therapies following trial discontinuation (Fig. 1C).

### Correlative studies

Paired core-needle tumor biopsy samples were successfully obtained from 20 patients both pre-treatment and on-treatment at the end of cycle 3 (see Additional file 1: method for details). An additional 11 pre-treatment biopsies did not have paired on-treatment biopsies, which in most cases was due to symptomatic progression without further staging. All biopsy samples, including unpaired (pre-treatment biopsy only) samples, were submitted for IHC, yielding 43 total samples evaluable by IHC. Paired samples from 17 patients contained evaluable tumor by IHC. A total of 38 samples were submitted for DNA methylation analysis, including paired samples from 15 patients with sufficient DNA for methylation analysis. A total of 36 samples were submitted for RNA sequencing, including paired samples from 16 patients.

### DNA methylation

**Fig. 3:**
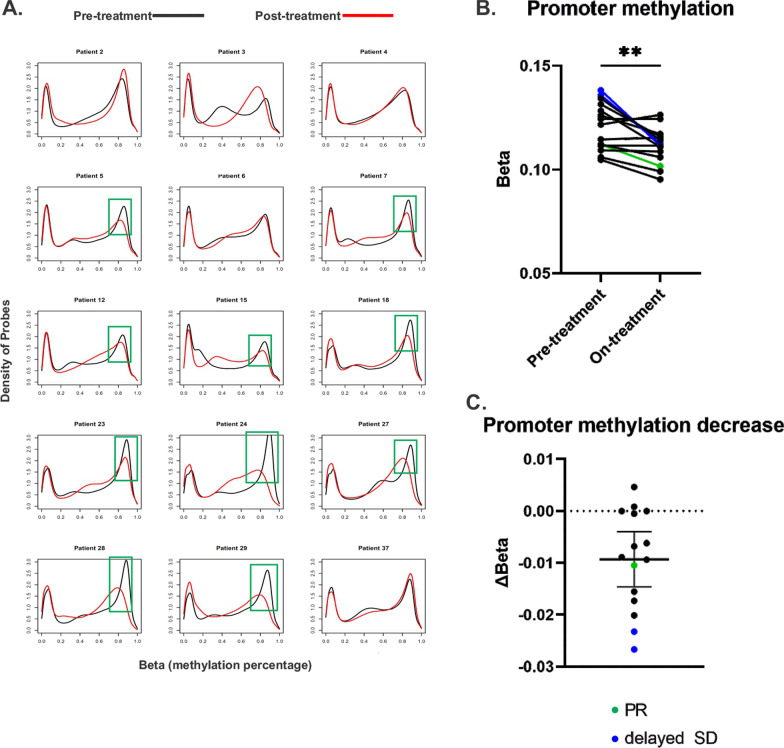
Tumor DNA methylation analysis. **A** Global DNA methylation beta plots for pre- and on-treatment samples are shown. X-axis (beta value) represents the ratio of methylated alleles to total alleles (methylated plus unmethylated) for each genomic site, and Y-axis represents the prevalence of the genomic sites with the corresponding beta values. Loci are unmethylated (beta values < 0.2). Green boxes highlight patients in whom many hypermethylated genomic loci decreased in methylation on-treatment compared to pre-treatment levels, indicating demethylation. **B** Average DNA methylation at promoter sites across the genome in pre- and on-treatment samples. **p < 0.005. **C** Difference between on-treatment and pre-treatment promoter site methylation for each patient, plotted with mean and 95% CI

DNA methylation analysis demonstrated relatively lower methylation of gene promoter regions and higher methylation globally and in transcription factor binding sites (TFBS) (Additional file 2: Fig. S3) at both baseline and after azacitidine treatment, consistent with previous findings [29]. Ten of 15 (67%) paired samples demonstrated decreases in methylation of many of the baseline hypermethylated loci on-treatment (Fig. 3A), shown by a shift of this peak to the left indicated global demethylation. Of these ten pairs showing decreased global methylation on-treatment, nine pairs also showed decreased gene promoter methylation (Additional file 2: Fig. S3). The average methylation of gene promoter regions decreased significantly on-treatment as compared to pre-treatment (Fig. 3B, C) for many patients. The one patient with a documented PR demonstrated a nearly identical average methylation profile in pre- and on-treatment analysis. However, his on-treatment biopsy contained no evaluable tumor for IHC analysis, suggesting the pre- and on-treatment comparison may not be valid in this patient. Overall, these results suggest a biochemical on-target effect of azacitidine on hypermethylated tumor DNA.

### RNA sequencing

**Fig. 4:**
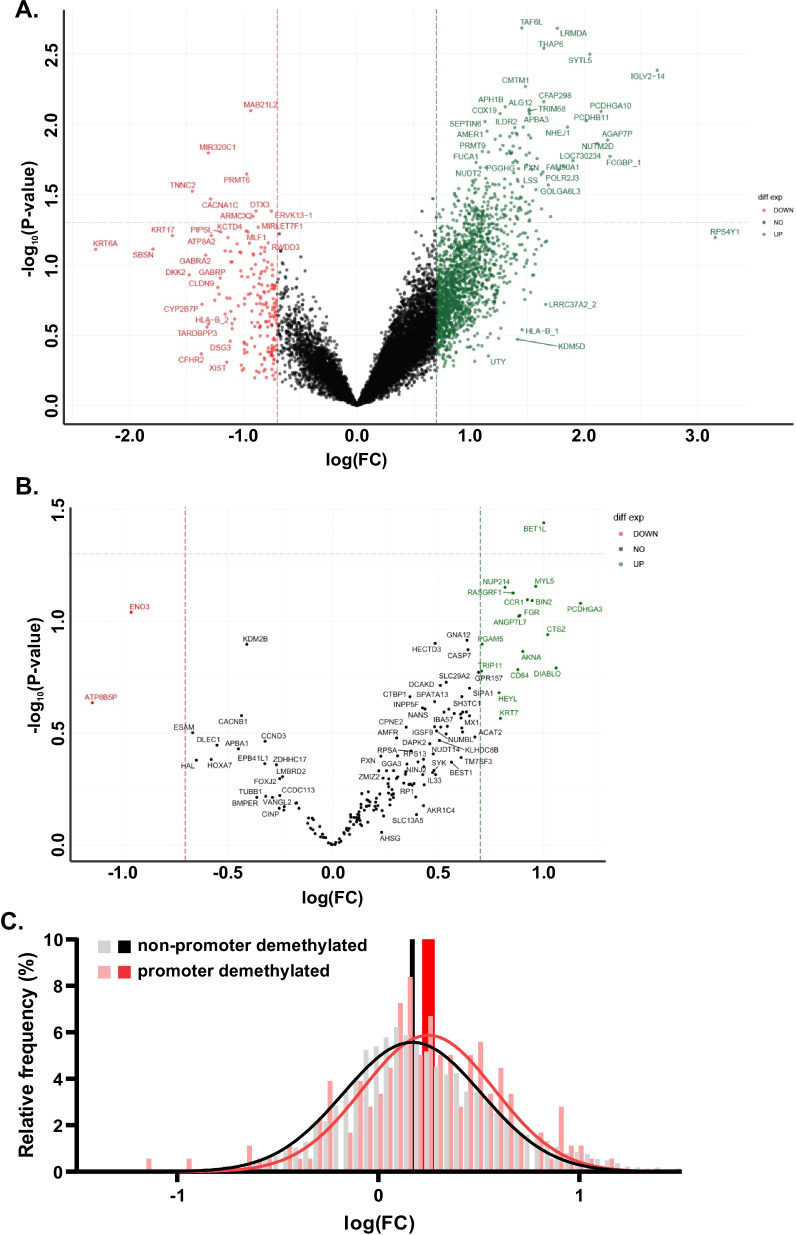
RNA sequencing. **A** RNA expression changes of all genes. Gray dotted line indicates *p* = 0.05, red and green dotted lines indicate − 0.7 and 0.7 log(fold change) decrease and increase, respectively. **B** RNA expression changes of genes that were determined to have the largest promoter region demethylation as determined by methylation analysis. Gray dotted line indicates *p* = 0.05, red and green dotted lines indicate − 0.7 and 0.7 log(fold change) decrease and increase, respectively. **C** Histogram and Gaussian distribution curves of the frequency of each degree of RNA expression change between pre- and on-treatment tumors. Gray and pink histogram bars indicate the individual frequencies of each level of RNA expression change in the non-promoter demethylated genes and promoter demethylated genes, respectively. Black and red lines are the Gaussian curves for the non-promoter demethylated genes and the promoter demethylated genes, respectively. Solid black and red bars indicate the 95% confidence intervals of the mean RNA expression change for the non-promoter demethylated genes and the promoter demethylated genes, respectively

We analyzed pre- and on-treatment tumors for correlative transcriptomic changes. Among all transcripts measured, the majority (71%) demonstrated increased expression on-treatment as compared to pre-treatment (Fig. 4A), although only a small proportion were statistically significant (1.4%). Due to the impact of gene promoter methylation on suppressing transcription, we further analyzed the subset of genes with the greatest promoter demethylation. In this subset, we observed a slightly larger majority of genes with increased on-treatment expression (78%, Fig. 4B). Consistent with this finding, genes which were demethylated most frequently in promoter regions demonstrated a greater average increase in expression than genes which were demethylated in the gene bodies (Fig. 4C). These findings indicate that our treatment combination led to global increases in gene expression, with a greater upregulation in the genes that underwent promoter demethylation.

### Immunologic biomarkers

Twelve patients (40%) had pre-treatment CD8^+^ TILs. Thirteen patients (43%) had no CD8^+^ TILs, and five patients’ biopsies were not evaluable (Fig. 1C). Twelve patients (40%) had the TIL-excluded phenotype in which CD8^+^ cells were present at the tumor/stroma interface but were absent within the tumor. Only one patient exhibited complete absence of TILs in both tumor and stroma. The one patient who had a PR had TILs of 2+, while the patient with SD had TILs of 1+. Of the other two patients who experienced delayed stabilization of disease, one had TILs of 3+ and the other had no evaluable tumor. Thus, all three patients with evaluable tumors who experienced clinical benefit (CB) from treatment had evidence of TILs within the tumor. In contrast, nine of 22 evaluable patients who did not benefit on treatment had TILs.

**Fig. 5:**
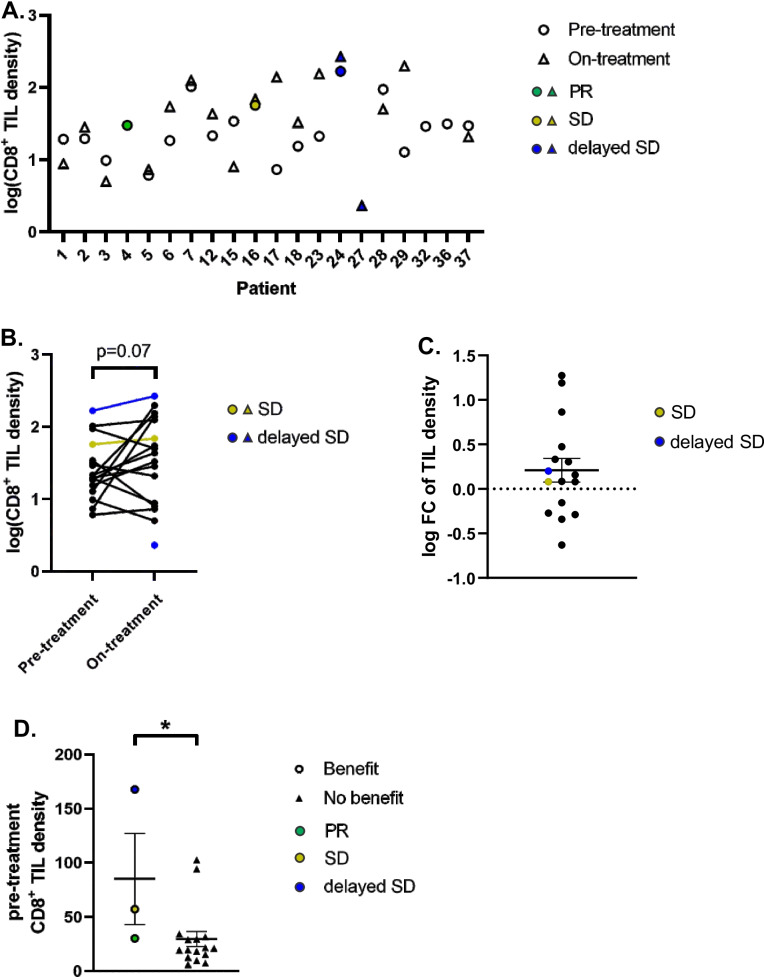
CD8^+^ TIL density. **A** TIL density in all evaluable pre-treatment and on-treatment samples, plotted on log scale. **B** Change in TIL density within individual pairs of tumors from pre- to on-treatment, plotted on log scale. Patient 4 (PR) had no assessable tumor on post-treatment biopsy. **C** Difference in TIL density within each patient’s tumor pairs, plotted with mean and SEM. **D** Pre-treatment TIL density in patients who benefitted versus patients who did not benefit from treatment, plotted with mean and SEM. *p < 0.05

A clinically validated digital pathology platform was utilized post hoc to quantify the CD8^+^ TIL density in patients’ tumors (Fig. 5A) [28]. The average TIL density trended higher in on-treatment samples as compared to pre-treatment samples (*p* = 0.07, Fig. 5B). Eleven of 16 (69%) of patients who produced an evaluable matched pair of biopsies demonstrated an increase in TIL density while on-treatment, while the remaining pairs (5 of 16, 31%) demonstrated a decrease in TIL density (Fig. 5C). These findings suggest that treatment with azacitidine and pembrolizumab may cause immune modulation and increase intra-tumoral TIL density. Pre-treatment TIL density of the tumors of patients who benefited from treatment was significantly higher than the TIL density of tumors from patients who did not benefit (Fig. 5D), suggesting that high pre-treatment TIL density may be associated with responsiveness to this treatment combination.

Tumor PD-L1 expression was detectable in three of 25 (12%) pre-treatment samples. The majority of the paired samples evaluable by IHC demonstrated increased tumor PD-L1 on-treatment (11 of 17 pairs, 65%), as assessed qualitatively. In contrast to tumor PD-L1, TIL PD-L1 was detectable in all 25 pre-treatment samples. TIL PD-L1 expression increased on seven of 17 pairs (41%) on-treatment as compared to pre-treatment.

The majority of tumors had detectable FOXP3^+^ Tregs in pre-treatment tumor (20 of 25 evaluable, 80%). All three patients who experienced clinical benefit and had evaluable biopsies, also had pre-treatment FOXP3^+^ Tregs at baseline, while 16 of 22 patients who experienced PD expressed FOXP3^+^ Tregs at baseline. The majority of on-treatment biopsies were also positive for Tregs (14 of 17 evaluable, 82%). Only four of 16 (25%) of the matched pairs of biopsies demonstrated a decrease in Tregs from pre- to on-treatment. Interestingly, while the patient who experienced PR did not have evaluable on-treatment tumor, the patient who experienced SD had no detectable Tregs on-treatment, which was a decrease from 1 + pre-treatment. Overall, these findings suggest that the treatment combination did not significantly modulate Treg infiltration in most tumors.

## Discussion

The main goal of this study was to investigate the ability of the DNMTi azacitidine to enhance the antitumor immune response of immune checkpoint therapy with pembrolizumab. To our knowledge, this was the first clinical trial conducted to investigate the efficacy, safety, and translational correlative science of the combination of DNMTi and immune checkpoint inhibitor in mCRC. Our treatment combination was well-tolerated with a manageable safety profile. The clinical efficacy of this combination was modest with only one PR and one SD being observed. However, in these two patients, treatment was continued for over 6 months. Another two patients remained on trial after initial restaging showed disease progression, and subsequently experienced progression-free intervals of 4 months and 2 months, respectively. These patients experiencing clinical benefit suggest that a subset of mCRC patients may indeed benefit from this treatment strategy. However, both the PFS and OS with this azacitidine–pembrolizumab combination are in line with patients managed on best supportive care in phase 3 trials of mCRC. Thus, based on the findings from this study, we are unable to recommend azacitidine and pembrolizumab for further clinical investigation in the treatment of patients with mCRC.

One potential reason for the inadequate clinical response was the use of a non-standard, low dose of azacitidine. Azacitidine is approved for use in MDS and AML at doses of 75 to 100 mg/m^2^, which equates to doses of 128 to 170 mg in a patient with a body surface area of 1.7 m^2^. We used a flat dose of azacitidine 100 mg, which is substantially lower than the standard approved doses. We chose this low dose because we wanted to test the hypothesis that prolonged exposure with a low concentration of DNMTi would be able to demethylate tumor DNA, as suggested by several key studies [20, 21]. While we did observe a positive pharmacodynamic effect in the form of tumor DNA demethylation, we likely did not benefit from a cytotoxic treatment effect from azacitidine.

Despite the minimal clinical activity observed with azacitidine-pembrolizumab combination, our study provides further insight into approaches combining epigenetic therapy with immunotherapy through the incorporation of paired tissue biopsies for examination of correlative biomarkers. Our analysis explored multiple pharmacodynamic correlations and hypothesized mechanisms of treatment with demethylating agents. We observed an overall reduction in global DNA methylation with a statistically significant decrease in the methylation of gene promoter regions in on-treatment tumors as compared to pre-treatment in the majority of patients with paired samples. While this is the expected biological outcome from treatment with a DNMTi, there is little evidence in previous studies for significant demethylation in tumors from patients with solid malignancies treated with demethylating agents. Taylor et al. recently published the results of the METADUR trial, which investigated the safety and activity of combination cc-486 (oral azacitidine), and durvalumab in advanced solid tumors [30]. Twenty-eight metastatic cancer patients, including 15 pMMR CRC patients were treated on this trial. Treatment with cc-486 + durvalumab resulted in similar survival times as our regimen, with no responders and two patients experiencing SD. Taylor et al. found modest tumor demethylation with cc-486 and suspected this to be the reason for lack of immune priming or clinical efficacy by their regimen. Our study appears to have found greater tumor demethylation and immune modulation, yet still produced modest clinical benefit. Aside from using subcutaneous azacitidine rather than oral azacitidine, another notable difference in our study was the collection of on-treatment biopsies at after three cycles of DNMTi treatment rather than after 2 cycles. This additional cycle of DNMTi could explain why our on-treatment biopsies appeared to show more tumor demethylation than was found by Taylor et al. Our demonstration of demethylation suggests that biological activity from azacitidine occurred as a result of this treatment. Other studies that have investigated changes in tumor DNA methylation have utilized more targeted approaches such as methylation analysis of specific genes [31]. To our knowledge, this is the first study to confirm the hypomethylating effect of a DNMTi in a solid tumor clinical trial utilizing a global DNA methylation analysis. Our results provide further evidence that standard dosing of DNMTi can, in fact, produce a demethylating effect in solid tumors.

We extended our analysis by using RNA sequencing to correlate gene expression changes with DNA demethylation, as well as test several mechanistic hypotheses for how DNMTi treatment may lead to immune priming. Globally, we observed greater upregulation of genes that experienced demethylation of their promoter regions, which supports our current biological understanding that promoter hypermethylation causes downregulation. To test our hypothesis for azacitidine-mediate immune priming, we performed gene set enrichment analysis using our RNAseq data. While on-treatment samples showed a trend toward increased interferon pathway expression (Additional file 2: Fig. S4A), the change was not statistically significant. We did not detect a change in total ERV expression. Two ERV genes (ERVFRD-1 and ERVH48-1, Additional file 2: Fig. S4B) demonstrated a trend toward increased expression on-treatment [32]. We also observed trends toward increased expression in several individual CTA genes (Additional file 2: Fig. S4C, D) [33]. In sum, our RNA sequencing analysis did not support a broad activation of either CTA expression or “viral mimicry” via ERV upregulation as mechanisms of low dose azacitidine-induced immune modulation. It is possible that significant biological signals exist within our data, but are covered by background noise due to patient and tissue heterogeneity. This obstacle could be overcome by the use of more granular high throughput sequencing methods, such as single cell RNA sequencing, in future studies.

CD8^+^ TILs have been shown to be one of the most critical effectors of antitumor immunity, and their presence in tumors is considered a positive signal for antitumor immune response [34]. In our study, CD8^+^ TILs were present in the tumors of the patients who experienced clinical benefit. Our post hoc quantification of CD8^+^ TIL density utilizing a novel digital pathology platform also suggested a potential correlation between higher pre-treatment TIL density and clinical benefit. TIL density, as measured by the proprietary Immunoscore digital IHC assay, has been validated as a biomarker predicting for survival and benefit from adjuvant chemotherapy for stages II-III CRC [35], although the significance of TIL density in mCRC remains unclear. Our study suggests that pre-treatment TIL density, as measured by digital pathology, may serve as a potential biomarker predicting for benefit from immunotherapy and warrants further study.

Our biomarker studies identified a potential immunomodulatory effect of the combination regimen, as demonstrated by the trend toward higher CD8^+^ TIL density in the on-treatment samples compared to pre-treatment samples. To determine if DNA demethylation from azacitidine treatment is associated with tumor-immune modulation, we examined the relationship between changes in CD8^+^ TIL density and changes in tumor methylation (Additional file 2: Fig. S5). We found a trend toward greater TIL increase and greater demethylation of all gene regions, promoter regions, and transcription factor binding sites (Additional file 2: Fig. S5A, B, C, Spearman's rank correlation coefficients − 0.37, − 0.34, and − 0.0.32; *p* values 0.13, 0.10, and 0.14, respectively). This trend is consistent with the primary scientific hypothesis that azacitidine induces tumor demethylation, which may then lead to immune modulation with increased TIL infiltration. While PD-1 inhibition monotherapy can cause immune modulation and TIL proliferation [36], all patients in our trial received the same dose of pembrolizumab and yet a fraction demonstrated significant increase in TILs. The association between demethylation and positive immune modulation suggests a true biological effect of azacitidine treatment in this trial, supporting preclinical studies that served as the scientific rationale for this combination approach [14, 20, 21, 37]. This is the first clinical study to demonstrate a relationship between demethylation and immunomodulation in a solid tumor, and suggests that DNA hypomethylating agents may indeed induce immunomodulation in patients.

Despite evidence for increased anti-tumor TILs in a subset of our patients, only one patient responded to treatment. This result is consistent with a recent study by Llosa et al., which found that although a high proportion of pMMR CRC had abundant CD8^+^ TILs but was still not responsive to pembrolizumab monotherapy [38]. This finding suggests that additional mechanisms of immune suppression must exist in pMMR CRC. We analyzed our RNA sequencing data for an increase in T-cell inflamed gene signature (Tinfl), which is considered a complementary marker of anti-tumor immune response [39, 40]. We observed 9 of 15 pairs as demonstrating increased Tinfl while on-treatment (Additional file 2: Fig. S5D), however the average change among all patients was not statistically significant. Furthermore, the change in Tinfl did not correlate with treatment benefit or TIL density change (data not shown). These findings further support the hypothesis that additional immune suppressive mechanisms exist within the TME which were not overcome by our treatment regimen. We observed the presence of FOXP3^+^ Tregs in most of our pre- and on-treatment tumors, consistent with other researchers finding that Tregs which are frequently present in various solid tumors function to suppress immunity [41]. Llosa et al. identified IL-17 and the Th17 subtype of T cells as a potential immune marker corresponding to poor response to checkpoint inhibition in pMMR mCRC [38]. If validated, the IL-17 pathway may then represent another potential therapeutic target.

The majority of mCRC is pMMR, and this group of patients is generally not responsive to PD-1/L1 blockade. Proposed mechanisms of immune evasion include tumor intrinsic factors such as low tumor mutational burden, and the microenvironment [8, 9]. The use of DNMTi as an immune priming agent is supported by abundant preclinical rationale [14], yet this approach has not yet previously demonstrated clinical efficacy. Together, both Taylor et al. and our study suggest that DNMTi and PD-1/L1 blockade is not an effective treatment combination for most patients with mCRC. The reasoning for this discrepancy between preclinical and clinical efficacy may be due in part to differences in the immune biology of the syngeneic murine tumor models often used to test immunomodulatory treatments. The MC38 syngeneic tumor model is often used to investigate developmental immunotherapeutics. However, transcriptional profiling has revealed this model to be more like dMMR CRC than pMMR CRC [42]. The CT26 model has similarly has been characterized as being like pMMR CRC. However, even this model has shown discrepant anti-tumor response when comparing analogous treatments in humans [24]. Targeting additional mechanisms, such as the aforementioned IL-17 pathway, FOXP3^+^ Tregs, or MDSCs, will likely be necessary in order to enhance anti-tumor immune responses. Our finding of tumor demethylation with increased CD8^+^ TIL infiltration may provide evidence supporting the use of DNMTi to complement these approaches.

Our clinical study has several important limitations. Our clinical trial was prematurely terminated due to the lack of clinical efficacy. Several patients were enrolled and received treatment, but subsequently experienced rapid progression prior to initial restaging. Immune checkpoint therapy often produces delayed responses when compared to cytotoxic chemotherapy, so that enrollment of patients with more aggressive and rapidly progressing disease may have led to these failures. This trial used RECIST 1.1 rather than irRECIST, thus some of the patients were deemed to have progression on the first restaging scans, but may have had pseudo-progression instead. By design, our study set the target ORR of 20% as the primary endpoint. However, recently approved agents for chemotherapy-refractory mCRC, including regorafenib and TAS-102, are associated with modest ORR of only 1–3% in FDA registration trials [2, 3], suggesting that either a lower ORR target or an alternative clinical efficacy endpoint such as PFS might have been a more appropriate endpoint for our trial. Limitations to the biomarker analysis must also be considered. Since DNA and RNA were extracted from the whole core biopsies rather than micro-dissected tumor tissues, the amount of tumor content versus normal or microenvironment tissue in each biopsy sample could have impacted our ability to compare pre- versus on-treatment changes. In the most extreme cases, a couple of on-treatment biopsies did not include any tumor tissue, as observed by IHC. The small number of patients who benefited from treatment limits our ability to determine the predictive value of any biomarker. Finally, the number of samples analyzed and the post-hoc nature of our RNA sequencing and TIL density quantification limit the significance of this analysis.

## Conclusions

In conclusion, the combination of pembrolizumab and azacitidine is safe and tolerable, but associated with only modest clinical activity in patients with chemotherapy-refractory mCRC. Despite the lack of clinical activity, we report that this combination regimen results in tumor DNA demethylation, RNA transcriptional increase, and immunomodulation. We present evidence for a potential link between DNA demethylation produced by treatment with hypomethylating agents and immunomodulation in a clinical context, supporting the preclinical hypothesis leading to this trial. While not sufficient for clinical response in most patients, this immunomodulatory approach may contribute to future strategies to overcome immune resistance in patients with mCRC.

## Supplementary Information

**Additional file 1**. Clinical trial protocol.

**Additional file 2**. Supplemental tables, figures, and methods.

## Data Availability

All datasets used and/or analyzed during the current study are available from the corresponding author on reasonable request.

## Abbreviations

CTA: Cancer testis antigen
CB: Clinical benefit
CR: Complete response
CT: Computed tomography
CRC: Colorectal cancer
CTA: Cancer-testis antigen
DNMTi: DNA methyltransferase inhibitor
ERV: Endogenous retrovirus
HDACi: Histone deacetylase inhibitor
IV: Intravenous
mCRC: Metastatic colorectal cancer
dMMR: Mismatch repair deficient
pMMR: Mismatch repair proficient
MDSC: Myeloid-derived suppressor cell
ORR: Objective response rate
OS: Overall survival
PR: Partial response
PD-1: Programmed cell death 1
PD-L1: Programmed cell death ligand 1
PFS: Progression-free survival
PD: Progression of disease
RECIST: Response evaluation criteria in solid tumors
RR: Response rate
SD: Stable disease
SC: Subcutaneous
TIL: Tumor-infiltrating lymphocyte
TRAE: Treatment-related adverse event
