# Supplementary material for: Pembrolizumab plus azacitidine in patients with chemotherapy refractory metastatic colorectal cancer: a single-arm phase 2 trial and correlative biomarker analysis
Source: Clin Epigenetics. 2022 Jan 6;14:3. doi: 10.1186/s13148-021-01226-y (PMC8740438; doi:10.1186/s13148-021-01226-y)
Supplement: Supplementary file 1 — Additional file 1. Clinical trial protocol. [file 13148_2021_1226_MOESM1_ESM.docx]

**Supplemental Tables**

**Supplemental Table 1.** Patient previous therapies and tumor mutation profile. Therapies listed in chronological order.

| **Pt** | **Previous therapies** | **KRAS** | **BRAF** | **PIK3CA** | **MMR** |
| --- | --- | --- | --- | --- | --- |
| 1 | FOLFOX + Bevacizumab, FOLFIRI + Bevacizumab | G12D | WT | WT | Unknown |
| 2 | FOLFOX + Bevacizumab, FOLFIRI + Cetuximab | WT | WT | WT | pMMR |
| 3 | FOLFOX + Cetuximab, FOLFIRI + Bevacizumab | WT | WT | WT | pMMR |
| 4 | FOLFIRI + Bevacizumab, FOLFOX, Temozolomide, Regorafenib | G12D | WT | WT | pMMR |
| 5 | FOLFOX, FOLFIRI + Bevacizumab, HIPEC, FOLFIRI + Bevacizumab , DC vaccine, FOLFOX, Irinotecan | G12D | WT | WT | pMMR |
| 6 | FOLFOX + Bevacizumab, Irinotecan + Bevacizumab, Regorafenib | G12S | Unknown | Unknown | MSS |
| 7 | FOLFOX + Bevacizumab, FOLFIRI + Bevacizumab, Regorafenib | G12R | WT | E542K | pMMR |
| 8 | FOLFOX + Bevacizumab, FOLFIRI + Bevacizumab, FOLFIRI + Cetuximab | WT | WT | Unknown | dMMR |
| 9 | FOLFOX, FOLFIRI + Cetuximab, Yttrium-90, HAI FUDR | G13D | WT | WT | pMMR |
| 10 | FOLFIRI + Bevacizumab, FOLFIRI + Ziv-aflibercept | G12 | Unknown | Unknown | Unknown |
| 12 | FOLFOX, FOLFIRI + Bevacizumab, Irinotecan + Cetuximab, Regorafenib, FOLFIRI + Ziv-aflibercept, FOLFOX | G12 | Unknown | Unknown | Unknown |
| 13 | FOLFOX + Bevacizumab, FOLFIRI + Bevacizumab | G12D | Unknown | Unknown | pMMR |
| 14 | FOLFIRI + Bevacizumab, FOLFOX + Bevacizumab, Yttrium-90, Regorafenib | G13D | WT | Unknown | MSS |
| 15 | FOLFOX + Bevacizumab, FOLFIRI + Ziv-aflibercept, Regorafenib | WT | WT | WT | Unknown |
| 16 | FOLFIRI, FOLFOX + Bevacizumab | G12L | Unknown | Unknown | Unknown |
| 17 | FOLFOX + Bevacizumab, Irinotecan + Cetuximab, Regorafenib | WT | WT | Unknown | pMMR |
| 18 | FOLFOX, Irinotecan, Regorafenib | G13D | WT | WT | pMMR |
| 21 | FOLFOX + Bevacizumab, Irinotecan + Panitumumab | WT | WT | WT | Unknown |
| 22 | FOLFOX + Bevacizumab, FOLFIRI + Bevacizumab, HAI FUDR | G12D | WT | WT | pMMR |
| 23 | FOLFIRI + Bevacizumab, FOLFOX + Bevacizumab | G60V | Unknown | Unknown | Unknown |
| 24 | FOLFOX + Bevacizumab, FOLFIRI + Bevacizumab | G12C | WT | Unknown | pMMR |
| 27 | FOLFOX + Bevacizumab, HIPEC, FOLFIRI + Ziv-aflibercept | G12V | WT | Unknown | unknown |
| 28 | FOLFOX + Bevacizumab, XRT, Capecitabine | WT | Unknown | Unknown | MSS |
| 29 | FOLFOX + Bevacizumab, FOLFIRI | G12D | Unknown | Unknown | pMMR |
| 32 | FOLFOX + Bevacizumab, FOLFIRI + Bevacizumab, FOLFIRI + Ziv-aflibercept, TAS-102 | G12D | WT | WT | pMMR |
| 33 | FOLFOX + Bevacizumab, FOLFIRI + Bevacizumab, FOLFIRI + Cetuximab | WT | WT | WT | MSS |
| 34 | FOLFOX + Bevacizumab, FOLFIRI + Bevacizumab, FOLFIRI + Panitumumab | WT | WT | WT | MSS |
| 35 | FOLFIRI + Bevacizumab, FOLFOX + Cetuximab, DC vaccine, FOLFIRI + Bevacizumab | WT | V600E | WT | pMMR |
| 36 | FOLFOX, FOLFIRI + Bevacizumab, Irinotecan + Panitumumab, Regorafenib, TAS-102 | G13D | WT | WT | pMMR |
| 37 | FOLFIRI + Bevacizumab, FOLFOX + Cetuximab, FOLFIRI + Panitumumab, TAS-102 | Unknown | Unknown | Unknown | Unknown |
| 39 | FOLFOX + Bevacizumab, Irinotecan + Cetuximab, Capecitabine + Ziv-aflibercept, Regorafenib | WT (NRAS G61R) | Unknown | Unknown | MSS |

**Supplemental Table 2.** IHC antibodies and assay conditions

| **Antibody** | **PD-L1** | **NY-ESO-1** | **CD3** | **CD4** | **CD8** | **FOXP3** | **PD-1** |
| --- | --- | --- | --- | --- | --- | --- | --- |
| **Source** | Merck | Sigma | Dako | Leica | eBioscience | eBioscience | Abcam |
| **Catalog #** | N/A | N2038 | A0452 | NCL-L-CD4-1F6 | 14-0085 | 14-4777-80 | ab52587 |
| **QML Lot #** | NT1511 | NT2018 | NT2025 | NT1641 | NT2723 | NT2722 | NT2721 |
| **Vendor Lot #** | N/A | 025M4801V | 20020069 | 6014648 | 1936473 | 1969041 | GR3174613-8 |
| **Isotype/Clone** | Mouse IgG1 | Mouse IgG1 | Rabbit IgG | Mouse IgG1 | Mouse IgG1 | Mouse IgG1 | Mouse IgG1 |
| **Clone** | Monoclonal  22C3 | Monoclonal  E978 | Polyclonal | Monoclonal  4B12 | Monoclonal  C8/144B | Monoclonal  236A/E7 | Monoclonal  NAT105 |
| **Dilution** | 2.0ug/mL | 1.5ug/mL | 1:250 | 5.0ug/mL | 0.375ug/mL | 3.0ug/mL | 2.0ug/mL |
| **Incubation Time** | O/N | 2 hours | 1 hour | 1 hour | O/N | O/N | O/N |
| **Pre-treatment** | SHIER 2 + Proteinase K | SHIER 8 + Proteinase K | SHIER 2, No Enzyme | SHIER 4 + Proteinase K | SHIER 2, No Enzyme | SHIER 2, No Enzyme | SHIER 2, No Enzyme |
| **Protocol** | MIPE | MIPE | MIP | MIPE | MIP | MIPE | MIP |
| **Detection System** | EnVision FLEX+ HRP Mouse | Polink-2 Plus HRP  Mouse | Polink-2 Plus HRP Rabbit | Polink-2 Plus HRP  Mouse | Polink-2 Plus HRP  Mouse | Polink-2 Plus HRP  Mouse | Polink-2 Plus HRP  Mouse |
| **Sub-Cellular Localization** | Plasma Membrane | Nucleus & Cytoplasm | Plasma Membrane | Plasma Membrane | Plasma Membrane | Nucleus | Plasma Membrane |

**Supplemental Figure Legends**

**Figure S1**. Clinical trial schema. Each cycle of therapy was 21 days long.

**Figure S2.** Patient 4 CT scans. CT scans were taken at baseline and at 12 weeks later. Patient 4 experienced partial response.

**Figure S3.** Average global methylation of (A) all genes and probes, (B) promoter sites only, and (C) transcription factor binding sites only. Y-axis is average beta value across all sites analyzed. Each point represents average global methylation of one patient’s pre- or on-treatment tumor. Patient 16 (SD) tumor biopsies were not assessable.

**Figure S4.** GSEA analysis. (A) GSEA comparing pre- and on-treatment biopsies using gene sets for antigen presentation (Ag_Pres), Interferon-a (INF_alpha), Interferon-y (INF_gamma), cytokines, inflammation. (B) Average expression changes of the 4 individual ERVs from Panda *et al* that demonstrated any appreciable expression in our RNA sequencing analysis^1^. (C) Average expression changes of individual CTAs from Yokoe *et al* with detectable expression in our RNA sequencing analysis^2^. (D) Average expression changes of individual CTAs from lncc with detectable expression in our RNA sequencing analysis^3^. All notable expression changes are indicated with pre- and on-treatment expression levels as well as p-values.

**Figure S5.** TIL density correlation with DNA demethylation. Correlation between change in CD8+ TIL density and change in DNA methylation of paired tumor samples. Spearman non-parametric r values were calculated for change in TIL versus change in (A) global, (B) promoter, or (C) transcription factor binding site demethylation. Only patients with pre- and on-treatment tumor samples that could be assessed for TIL density as well as DNA methylation were included. All correlation coefficients were statistically non-significant (p = 0.13, 0.10, and 0.14 for A, B, and C, respectively). (D) Change in T-cell inflamed gene signatures of pre- versus on-treatment biopsies for each patient with analyzable paired biopsies.

**Supplemental Methods**

**Immunohistochemistry**

Immunohistochemical (IHC) staining for PD-L1 used a mouse monoclonal anti-PD-L1 antibody (clone 22C3, Merck, Palo Alto, CA) capable of detecting PD-L1 in formalin-fixed, paraffin-embedded (FFPE) specimens. In brief, FFPE tissue blocks were cut 4 μm thickness, and sections mounted onto positively-charged glass slides. Slides were baked (60°C, dry heat, for 45 minutes) immediately prior to use. Tissue sections were de-waxed using organic solvents (xylene, 100%, four changes), and an alcohol series (100%, 70%, 30% ethanol) descending to distilled water.

Low pH Target Retrieval Solution (Dako Cat. No. S1700) was used for the unmasking of the 22C3 antigen/epitope of PD-L1 with a commercial steamer (20 minutes above 90°C) as heat source. Upon cooling slides (5 minutes), retrieval of the 22C3 antigen/epitope of PD-L1 was further advanced using Proteinase K (Dako Cat. No. S3020, 1:160 dilution in EnVision™ FLEX+ wash buffer, 10 minutes). Primary antibody incubation (PD-L1 mAb clone 22C3, 2μg/mL) was performed off-platform for 16 ± 1 hours in a dark humidified chamber. Primary Antibody Diluent (Dako Cat. No. S0809) was used to prepare working dilutions of PD-L1 (22C3) and isotype positive and negative control antibodies (Dako Cat. No. M0755 CD20cy and R&D Systems Cat. No. MAB002 Mouse IgG1κ). Target recognition for PD-L1 (22C3) and the site of antigen-primary antibody interaction in FFPE sections used all wash, block, and signal amplification/detection reagents from the EnVision FLEX+ HRP-Polymer kit (Dako Cat. No. K8012). EnVision™ FLEX+ Mouse Linker (15 minutes), EnVision™ FLEX+ HRP-polymer (25 minutes), EnVision™ FLEX+ DAB Chromogen (10 minutes), nickel chloride (Ni2+Cl2) DAB Enhancer (10 minutes), with hematoxylin counterstain (1/5 dilution, 1 minute), completed the assay protocol. Between all incubation steps, slides were extensively washed with EnVision™ FLEX+ wash buffer.

At the conclusion, slides were rinsed in distilled water, dehydrated in an alcohol series (95%, 100% ethanol) and organic solvent (xylene, 100%, four changes), and permanently coverslipped for storage and interpretation.

These steps were automated with a TechMate Instrument (Roche Diagnostics) running QML workmate software v3.96. This automated platform uses a capillary gap process for all reagent changes, up to and including counterstaining, and intervening buffer washes. All steps were carried out at room temperature (25°C).

The target antigen was identified by light microscopy. Nuclei were counterstained using hematoxylin to further assess cell and tissue morphology. Additional IHC markers (NY-ESO-1, CD3, CD4, CD8, FOXP3, PD-1) were analyzed in a similar fashion as PD-L1, with the following modifications:

SHIER 2 (Citrate-based, pH 6.0-6.2), SHIER 4 (Tris-EDTA, pH 9), and SHIER 8 (AR10, pH10) solutions was used for unmasking antigens/epitopes using a commercial steamer (20 minutes above 97°C) as a heat source. After heat-induced epitope retrieval, the process steps were automated using a TechMate Instrument (Roche Diagnostics) running QML workmate software v3.96.

Reagent Manufacturing Buffer (RMB) with Goat Serum was used to prepare working dilutions of all primary antibodies, species-match positive controls, and isotype-match negative controls. Target recognition at the site of antigen-primary antibody interaction in FFPE sections uses detection reagents from Polink-2 Plus HRP kits from GBI Labs designed for detection of Mouse or Rabbit primary antibodies.

For PD-L1 and NY-ESO scoring, a modified H-score was used. The modified H-score was calculated based on the percent of tumor with membrane-specific staining at four different intensity levels: negative (<1), low (1+), moderate (2+), and high (3+). Each staining level was assigned a percent of tumor score from one of the following: 0, 1, 2, 5, 10, 15, 20, 30, 40, 50, 60, 70, 80, 85, 90, 93, 95, 99, or 100%. Each intensity level was multiplied by its corresponding staining percentage level and then all products were summed to arrive at the final modified H-score.

For immune biomarker scoring, 20x fields of the entire tissue specimen were reviewed with the aid of accompanying H&E stained slides. Scores of 0-3 were assigned based on the following: 0 (no positive immune cells, <1 cell/20X field), 1 (low density, 1-10 positive immune cells/20X field), 2 (moderate density, 11-20 positive immune cells/20X field), and 3 (high density, >20 positive immune cells/20X field).

Refer to supplemental table 2 for antibody specifications and IHC assay conditions.

**Research Biopsies**

In the interest of maximizing benefit and data sharing from a clinical trial which mandated research biopsies both prior to treatment and on-treatment, we report the following characteristics of the biopsies. For the purpose of this report, N = 31+20 represents the combination of total pre-treatment and on-treatment biopsies in the intention to treat population. This number also includes one patient who underwent enrollment and pre-treatment biopsy but withdrew prior to treatment. Patient adverse events related to the biopsy procedures themselves were evaluated by chart review of the procedural notes and subsequent clinic notes for each patient.

|  | **Patient biopsies (N = 51)** | **% biopsies** |
| --- | --- | --- |
| Pre-treatment biopsies | 31 | - |
| On-treatment biopsies | 20 | - |
| Total biopsies | 51 | 100% |
| No evaluable tumor for all IHC markers | 9 | 18% |
| Not evaluable for at least one IHC marker | 10 | 20% |
| Peri-procedural adverse events | 0 | 0% |

**Supplemental References**

1. Panda, A., Cubas, A. A. de, Stein, M., Riedlinger, G., Kra, J., Mayer, T., *et al.* Endogenous retrovirus expression is associated with response to immune checkpoint blockade in clear cell renal cell carcinoma. *JCI Insight* **3**, (2018).

2. Yokoe, T., Tanaka, F., Mimori, K., Inoue, H., Ohmachi, T., Kusunoki, M., *et al.* Efficient identification of a novel cancer/testis antigen for immunotherapy using three-step microarray analysis. *Cancer Res.* **68**, 1074–1082 (2008).

3. Almeida, L. G., Sakabe, N. J., de Oliveira, A. R., Silva, M. C. C., Mundstein, A. S., Cohen, T., *et al.* CTdatabase: A knowledge-base of high-throughput and curated data on cancer-testis antigens. *Nucleic Acids Res.* **37**, (2009).
