# Supplementary material for: Pembrolizumab plus azacitidine in patients with chemotherapy refractory metastatic colorectal cancer: a single-arm phase 2 trial and correlative biomarker analysis
Source: Clin Epigenetics. 2022 Jan 6;14:3. doi: 10.1186/s13148-021-01226-y (PMC8740438; doi:10.1186/s13148-021-01226-y)
Supplement: Supplementary file 2 — Additional file 2. Supplemental tables, figures, and methods. [file 13148_2021_1226_MOESM2_ESM.pdf]

Figure S1.

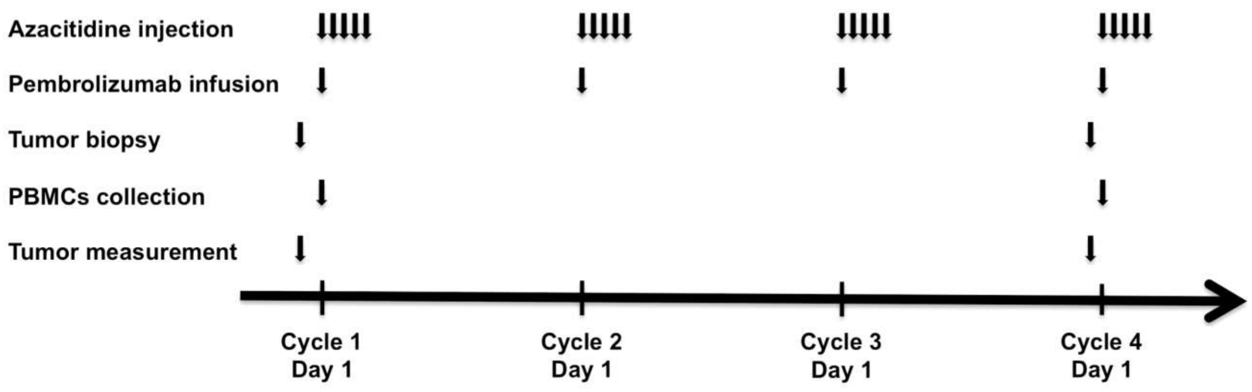
Azacitidine injection
Pembrolizumab infusion
Tumor biopsy
PBMCs collection
Tumor measurement
Cycle 1
Day 1
Cycle 2
Day 1
Cycle 3
Day 1
Cycle 4
Day 1

Figure S2.

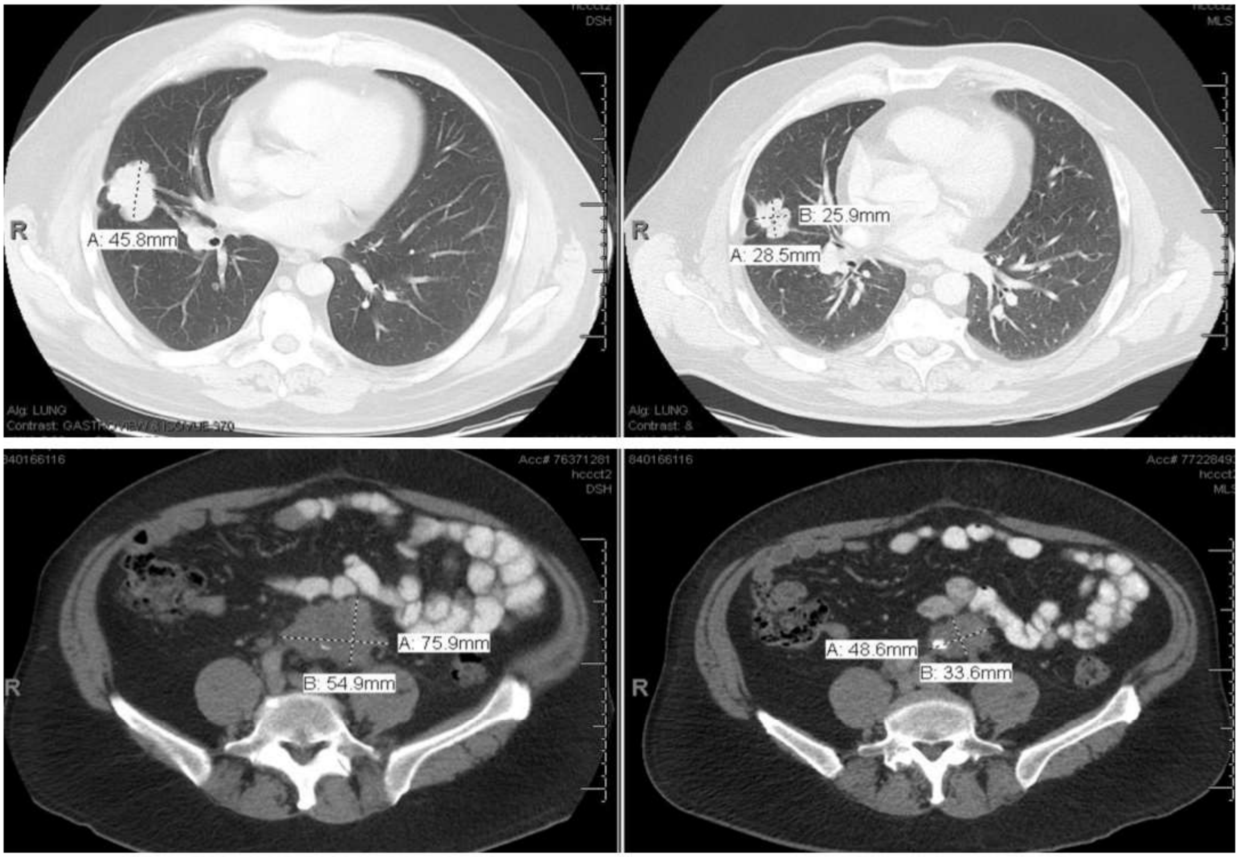

At the baseline
(before cycle 1 day 1)

At the end of cycle 4 (12 weeks
from cycle 1 day 1)
→ 32% reduction of target lesions

Figure S3.

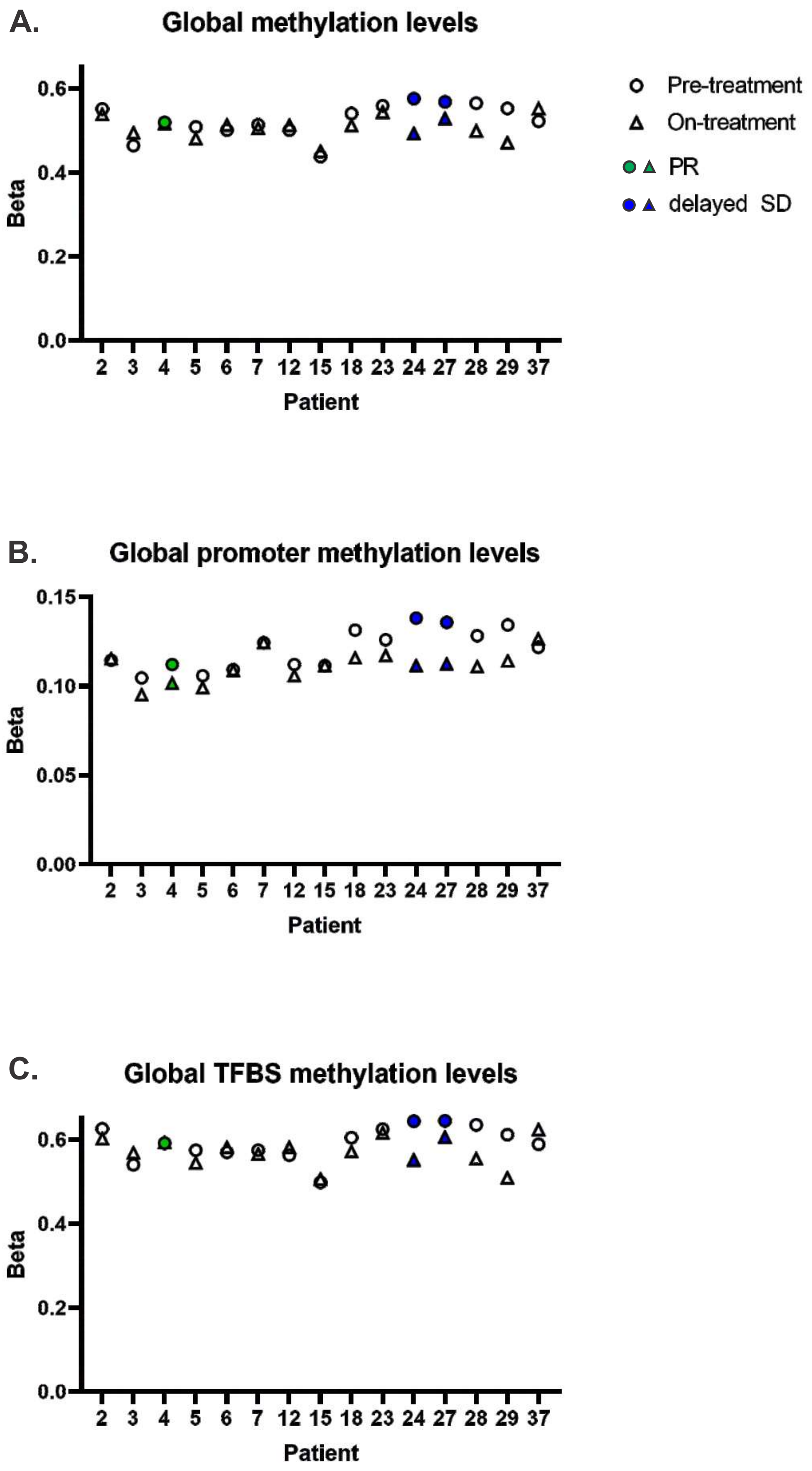
A.
Global methylation levels
Beta
0.0
0.2
0.4
0.6
2 3 4 5 6 7 12 15 18 23 24 27 28 29 37
Patient
Pre-treatment
On-treatment
PR
delayed SD
B.
Global promoter methylation levels
Beta
0.00
0.05
0.10
0.15
2 3 4 5 6 7 12 15 18 23 24 27 28 29 37
Patient
C.
Global TFBS methylation levels
Beta
0.0
0.2
0.4
0.6
2 3 4 5 6 7 12 15 18 23 24 27 28 29 37
Patient

Figure S4.

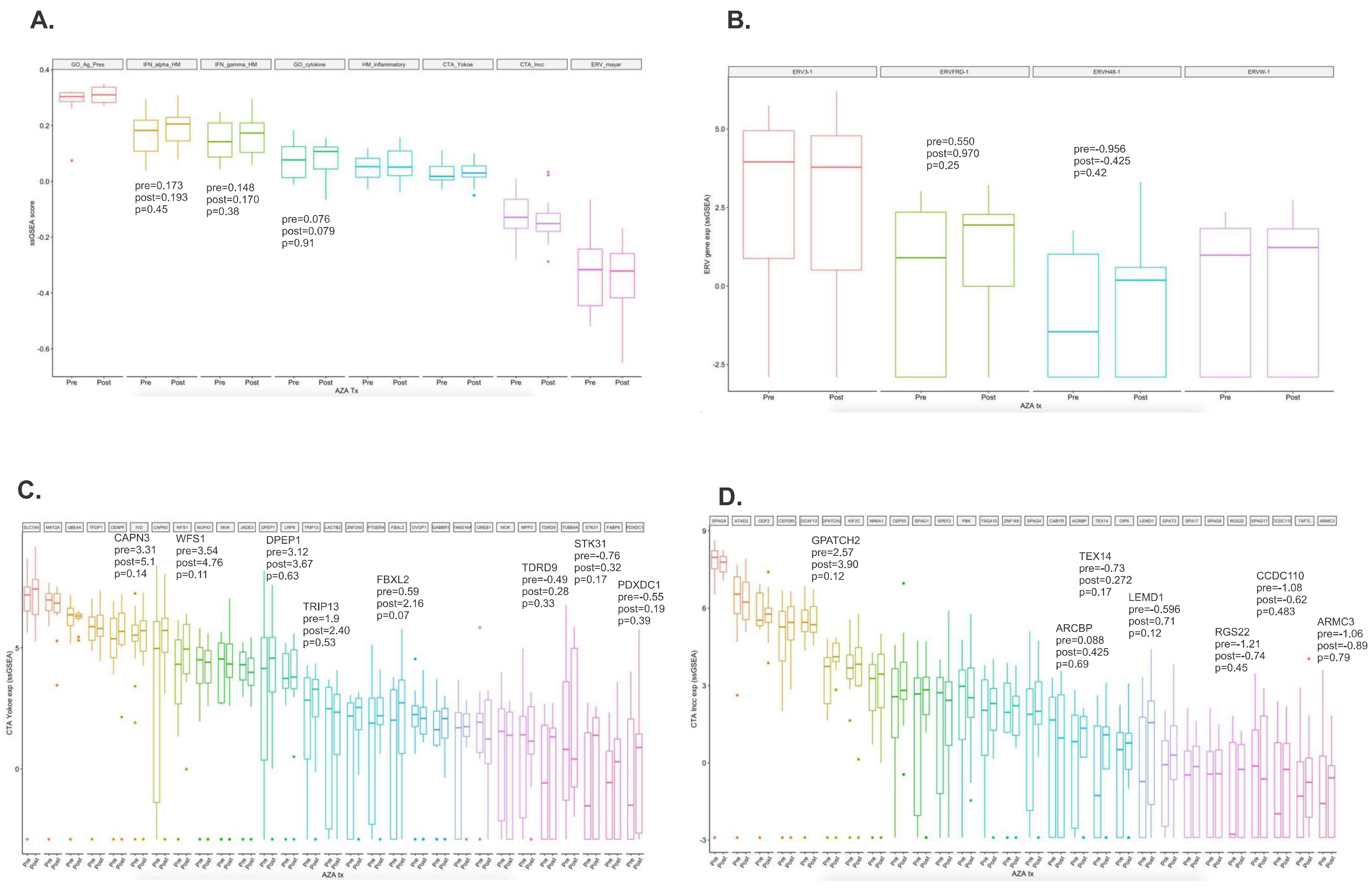
A.
GO_Ag_Pres
IFN_alpha_HM
IFN_gamma_HM
GO_cytokine
HM_inflammatory
CTA_Yokoe
CTA_lncc
ERV_mayar
ssGSEA score
pre=0.173
post=0.193
p=0.45
pre=0.148
post=0.170
p=0.38
pre=0.076
post=0.079
p=0.91
AZA Tx
B.
ERV3-1
ERVFRD-1
ERVH48-1
ERVW-1
ERV gene exp (ssGSEA)
pre=0.550
post=0.970
p=0.25
pre=-0.956
post=-0.425
p=0.42
AZA tx
C.
CAPN3
pre=3.31
post=5.1
p=0.14
WFS1
pre=3.54
post=4.76
p=0.11
DPEP1
pre=3.12
post=3.67
p=0.63
TRIP13
pre=1.9
post=2.40
p=0.53
FBXL2
pre=0.59
post=2.16
p=0.07
TDRD9
pre=-0.49
post=0.28
p=0.33
STK31
pre=-0.76
post=0.32
p=0.17
PDXDC1
pre=-0.55
post=0.19
p=0.39
CTA Yokoe exp (ssGSEA)
AZA tx
D.
GPATCH2
pre=2.57
post=3.90
p=0.12
TEX14
pre=-0.73
post=0.272
p=0.17
ARCBP
pre=0.088
post=0.425
p=0.69
LEMD1
pre=-0.596
post=0.71
p=0.12
RGS22
pre=-1.21
post=-0.74
p=0.45
CCDC110
pre=-1.08
post=-0.62
p=0.483
ARMC3
pre=-1.06
post=-0.89
p=0.79
CTA lncc exp (ssGSEA)
AZA tx

Figure S5.

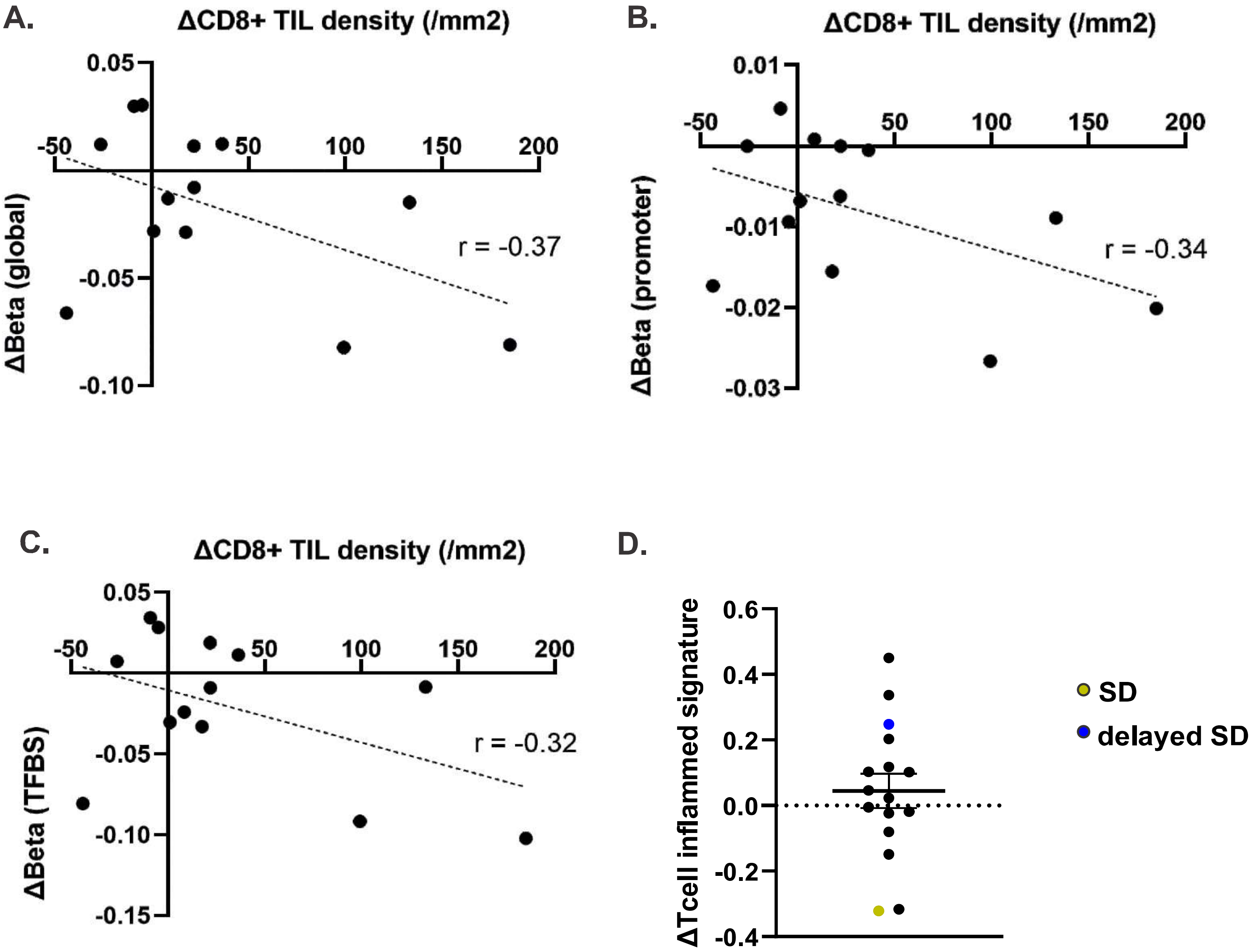
A.
ΔCD8+ TIL density (/mm2)
ΔBeta (global)
0.05
-0.05
-0.10
-50
50
100
150
200
r = -0.37
B.
ΔCD8+ TIL density (/mm2)
ΔBeta (promoter)
0.01
-0.01
-0.02
-0.03
-50
50
100
150
200
r = -0.34
C.
ΔCD8+ TIL density (/mm2)
ΔBeta (TFBS)
0.05
-0.05
-0.10
-0.15
-50
50
100
150
200
r = -0.32
D.
ΔTcell inflammed signature
0.6
0.4
0.2
0.0
-0.2
-0.4
SD
delayed SD
